# Correction of vibration artifacts in DTI using phase-encoding reversal (COVIPER)

**DOI:** 10.1002/mrm.23308

**Published:** 2012-09

**Authors:** Siawoosh Mohammadi, Zoltan Nagy, Chloe Hutton, Oliver Josephs, Nikolaus Weiskopf

**Affiliations:** 1 Wellcome Trust Centre for Neuroimaging, UCL Institute of Neurology, University College London, United Kingdom

**Keywords:** partial Fourier imaging, table vibration, blip-up blip-down, *k*-space echo shift

## Abstract

Diffusion tensor imaging is widely used in research and clinical applications, but still suffers from substantial artifacts. Here, we focus on vibrations induced by strong diffusion gradients in diffusion tensor imaging, causing an echo shift in *k*-space and consequential signal-loss. We refined the model of vibration-induced echo shifts, showing that asymmetric *k*-space coverage in widely used Partial Fourier acquisitions results in locally differing signal loss in images acquired with reversed phase encoding direction (blip-up/blip-down). We implemented a correction of vibration artifacts in diffusion tensor imaging using phase-encoding reversal (COVIPER) by combining blip-up and blip-down images, each weighted by a function of its local tensor-fit error. COVIPER was validated against low vibration reference data, resulting in an error reduction of about 72% in fractional anisotropy maps. COVIPER can be combined with other corrections based on phase encoding reversal, providing a comprehensive correction for eddy currents, susceptibility-related distortions and vibration artifact reduction. Magn Reson Med, 2012. © 2011 Wiley Periodicals, Inc.

Diffusion tensor imaging (DTI) allows for noninvasive imaging of water diffusion (1–4), an important marker for brain anatomy and physiology. In addition to its wide spread use in neuroscience research (5–9), DTI has become an essential diagnostic tool in stroke and neurodegenerative disease due to its sensitivity to microstructural and physiological changes (10–16).

However, obtaining reliable DTI data and drawing meaningful inference from them are more challenging than for conventional imaging modalities such as T1- or T2-weighted images, because in diffusion MRI the contrast depends on the molecular water diffusion at the micrometer scale (17–20). To achieve the required diffusion sensitization strong imaging gradients need to be applied, which usually entails a high first-order gradient moment. The large first moment makes the DTI sequence sensitive to brain-tissue movements [e.g., due to human physiology (21, 22) or instrumental vibration (23, 24)], resulting in a substantial echo shift in *k*-space locally where strong movement occurs. If this echo shift exceeds the *k*-space acquisition window, severe signal loss occurs.

Instrumental vibration is a recently discovered source (23) of movement-related signal-loss in DTI affecting multiple research sites and different types of scanners (20, 25–28). Strong diffusion gradients excite low-frequency mechanical resonances of the MR system (29). This leads to a patient table vibration, which can be transferred to the subject's brain (24). Movement-related signal-loss in diffusion-weighted (DW) images can reduce the diagnostic value of DW imaging (23), bias the estimation of the diffusion tensor (21, 23, 25, 27, 30), reduce the comparability of different quantitative DTI indices and hinder emerging diffusion techniques, which increasingly rely on high diffusion weighting (31–33).

Retrospective correction methods have been established for well-known artifacts such as eddy currents (e.g., Refs.^34^ and^35^) and susceptibility-related distortion (e.g.,Refs. 36 and^37^). In contrast, vibration artifacts in DTI have been explored and reported in the literature only recently (20, 23, 25, 27, 28). The goal of this study was to retrospectively correct vibration-induced signal loss motivated by a similar well established approach for reducing susceptibility-induced signal loss due to echo shifts in gradient echo echo-planar imaging (38), which is based on the concept of acquiring two data sets with opposite phase encoding (PE) direction (39, 40). Although the acquisition and combination of data with reversed PE direction has already been established for DTI to correct for susceptibility and eddy current induced distortion (37, 41–44), it needs to be modified for reduction of vibration artifacts.

We have developed a simple method for correction of vibration artifacts in DTI using phase-encoding reversal (COVIPER) by combining two images with reversed PE direction, each weighted by a function of its local tensor-fit error. To test the proposed method, we compared the fractional anisotropy (FA) and tensor-fit error of COVIPER corrected images with images combined using the conventional arithmetic mean (37, 41), and with reference images that contained negligible vibration artifacts.

## Materials and Methods

### Theory

Motion during the diffusion-weighting period may shift the echo center towards the edge of *k*-space in PE direction (Fig 1) and lead to loss of the DW signal, as has been demonstrated for linear rigid-body motion (45). For linear rigid-body motion the shift of the echo center **Δk** only depends on rotational but not on the translational movement (45, 46):

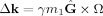
1 where *γ* is the gyromagnetic ratio, **Ω** is the angular velocity vector, 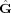
a dimensionless unit vector in the diffusion gradient direction and *m*_*1*_ is the first moment of the gradient. Note that the first order gradient moment depends on the gradient waveform and thus might vary between diffusion sequences (see e.g., Ref.^47^).

**Figure 1:**
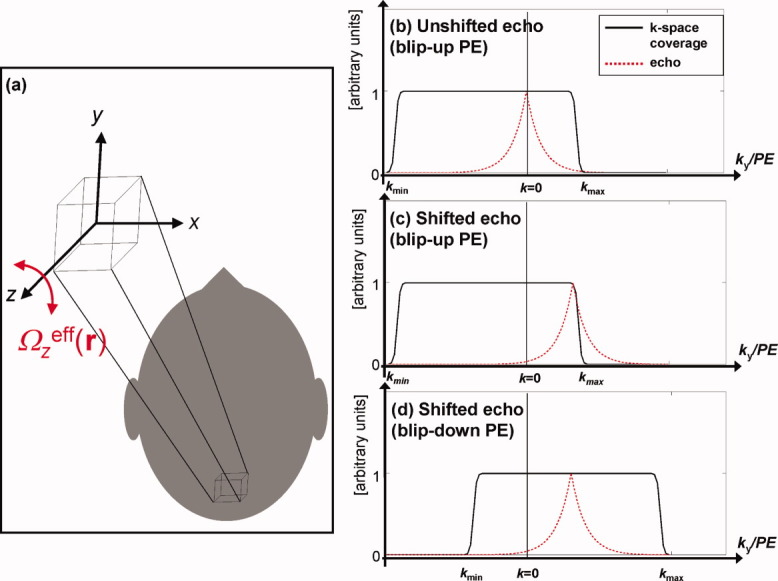
Schematic diagrams showing (a) vibration-induced rotational in-plane movement 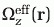
(r) of the brain-tissue, (b–d) the *k*-space coverage (black curve) and the echo (red dashed curve). The data are assumed to be sampled asymmetrically over the range [*k*_min_, *k*_max_] due to Partial Fourier imaging in phase-encoding (PE) direction. b: In the absence of motion, the echo center is at *k*_*y*_ = 0. c: Vibration-induced rotational movement of the brain-tissue might shift the echo center towards the shorter *k*-space edge (here *k*_max_) for a given PE direction (here blip-up) and lead to signal-loss. d: When reversing the polarity of the PE direction (blip-down), the acquisition window is shifted in such a way that short and long *k*-space edges are swapped. In this case, the echo center can be fully sampled and the signal will be preserved. Note that in other parts of the brain the echo center might be shifted towards the negative *k*-space edge, leading to signal-loss in the blip-down images. In this case, the above model is still valid but in a reversed manner. [Color figure can be viewed in the online issue, which is available at wileyonlinelibrary.com.]

Usually movement-induced signal-dropout is caused by shifting the echo center in PE direction (e.g., Refs.^23^ and^45^), because the *k*-space is often only partially covered in Partial Fourier imaging. Previous studies suggested that mechanical vibration of the scanner primarily causes nonrigid rotational movement of the brain tissue in the transverse plane, which is relatively slow, varying across the brain (24). Motivated by those measurements, we assumed that the spatial derivative of the rotational in-plane movement could be neglected. Giving this approximation and assuming that the PE direction is applied along the anterior-posterior (*y*) direction the shift in the *k*_*y*_-direction can be described as follows:

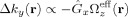
2 where 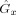
is the *x* component of the diffusion gradient vector and 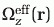
(**r**) is the *z* component of the effective angular velocity vector at voxel position **r** due to local rotational in-plane movement (Fig 1a). Note that 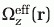
(**r**) in Eq. 2 is time-averaged, i.e., it has been integrated over the diffusion weighting time.

According to Eq. 2, the local effective angular velocity might be strong enough to shift the echo center out of the *k*-space acquisition window (Fig 1c). Since the central echo carries the main signal power, severe signal dropout will occur in this case (38, 48). The acquisition window can be shifted by reversing the PE direction as shown in Fig 1c,d, to capture the shifted main echo. If two images with reversed PE direction (subsequently denoted as blip-up and blip-down data set) are acquired, the signal lost in one of these images may be recovered in the other if the shift Δ*k*_*y*_(**r**) does not exceed the nominal *k*-space window of both images, i.e.:

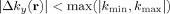
3

### Subjects

Three healthy volunteers (two female and one male) participated in the study after giving written informed consent, approved by the local ethics committee.

### Data Acquisition

For data acquisition a TIM Trio 3T scanner (Siemens Healthcare, Erlangen, Germany) with a single channel transmit-receive RF head coil was used. For each subject an axial, dual echo, gradient echo FLASH sequence was measured for B0 static magnetic field mapping (49, 50). DTI data were acquired with an in-house developed DTI sequence (51, 52) based on the twice-refocused spin echo diffusion scheme of (53) and using the following parameters: 60 DW images with spherically distributed diffusion-gradient directions (54), six non-DW images, matrix 96 × 96, 60 slices, 2.3 mm isotropic resolution, 5/8 Partial Fourier in PE direction using zero filling reconstruction, TE = 86 ms.

For each subject four DTI datasets were acquired (Table 1): (a) a time efficient short slice repetition time (slice TR = 140 ms; volume TR = 8.4 s) using the default PE direction (blip-up DTI data set, DTI_1_^+^), (b) the same acquisition as in (a) but using reversed PE direction (blip-down DTI data set, DTI_1_^−^), (c and d) the same measurement as in (a) and (b) but using a longer TR (slice TR = 170 ms, volume TR = 10.2 s, DTI_2_^+^ and DTI_2_^−^). Due to the short TR, datasets (a) and (b) were prone to vibration artifacts. Increasing the TR in datasets (c) and (d) made the data less affected by vibration artifacts, because the damped low-frequency mechanical vibration of the patient table induced by the diffusion gradients fade with increasing TR (see Refs.^23^ and^29^). As a result, the measurements (c) and (d) were used as the reference dataset.

**Table 1:** Slice Repetition Time (TR) and Phase-Encoding (PE) Direction for Each of the Four Different DTI Data Sets

	TR (ms)	PE direction
DTI_1_^+^	140	Blip-up
DTI_1_^−^	140	Blip-down
DTI_2_^+^	170	Blip-up
DTI_2_^−^	170	Blip-down

### Data Preprocessing

All analysis steps were performed using SPM8 [http://www.fil.ion.ucl.ac.uk/spm; (55)] and in-house software written in MATLAB (version 7.11.0; Mathworks, Natick, MA). First, all four DTI datasets were corrected for motion and eddy current effects (35). Next, the dual gradient echo FLASH data were processed to create a voxel displacement map (50). Each of the four DTI datasets was corrected for susceptibility-induced geometric distortion using this displacement map (23, 50). Finally, to reduce residual misalignments between the blip-up and blip-down datasets, the first non-DW image of each blip-down DTI dataset was registered to the corresponding image of the blip-up DTI dataset and the resulting 12-parameter affine transformation was applied to the other images of the blip-down dataset.

### COVIPER Method

The COVIPER method is a two-step procedure. In the first step, the diffusion tensor (**D**) as well as the residual error of the tensor fit (ε) were calculated for all four DTI datasets of each subject using the standard diffusion tensor formalism (56). In the second step, the apparent diffusion coefficients (56) of the blip-up (+) and blip-down (−) DTI data were combined using a local weighted-sum approach:


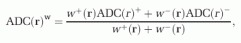
4 where the spatially varying weighting *w*^*j*^(***r***) is given by a Lorentzian function of the error in the tensor fit:


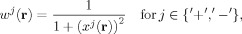
5 where *x*^*j*^(**r**) is the normalized maximum-norm of the tensor-fit error **ε**^*j*^ = [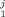
(**r**),…, 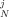
(**r**)] over all applied diffusion directions (*N* = number of directions), i.e., 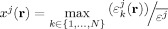. The corresponding normalization factor 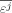 is given by the spatially averaged root-mean-square of the tensor-fit error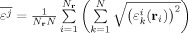, with *N*_**r**_ being the number of voxels in the brain 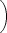.

### Assessment of COVIPER

Four types of FA images were calculated from the diffusion tensor of the affected (DTI_1_^+^ and DTI_1_^−^) and reference (DTI_2_^+^ and DTI_2_^−^) datasets: two directly (FA^+^_1,2_ and FA^−^_1,2_) and the other two using their combination (arithmetic mean of the images, FA^mean^_1,2_, and weighted sum of the images, FA^w^_1,2_). To remove residual anatomical differences due to movement, each FA image of the affected datasets (DTI_1_^±^) was registered to the corresponding FA image of the reference dataset (DTI_2_^±^). Next, the vibration-induced bias in FA (ΔFA^bias^) and its corrections (ΔFA^mean^ and ΔFA^w^) were assessed by means of a root-mean-square FA difference between affected and reference data:

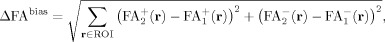
6a and 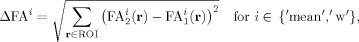
6b with ROI being a region-of-interest that was affected by vibration artifacts and calculated from the tensor-fit error map of the short-TR dataset (Table 1). Finally, to assess miscellaneous effects of the proposed correction method (ΔFA^misc^), the root-mean-square FA difference between the arithmetic-mean and weighted-sum combination was calculated for the reference data with reduced vibration artifacts, i.e., no correction effect for vibration artifacts was expected: 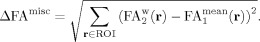
6c

## RESULTS

Figure 2a shows an example of the signal-dropout due to vibration in an axial slice of a diffusion-weighted image (top row) as well as the corresponding shift of the echo in *k*-space (middle row) and its *k*-space signal profile along the dashed line (bottom row). The signal-dropout was most apparent when the echo center was shifted towards the shorter *k*-space edge (red arrow). For the reference DTI data (Fig 2b), which were acquired using a longer slice TR = 170 ms to reduce the vibration effects, the echo center was only marginally shifted and no signal-dropout was visible in the diffusion-weighted image of either blip direction.

**Figure 2:**
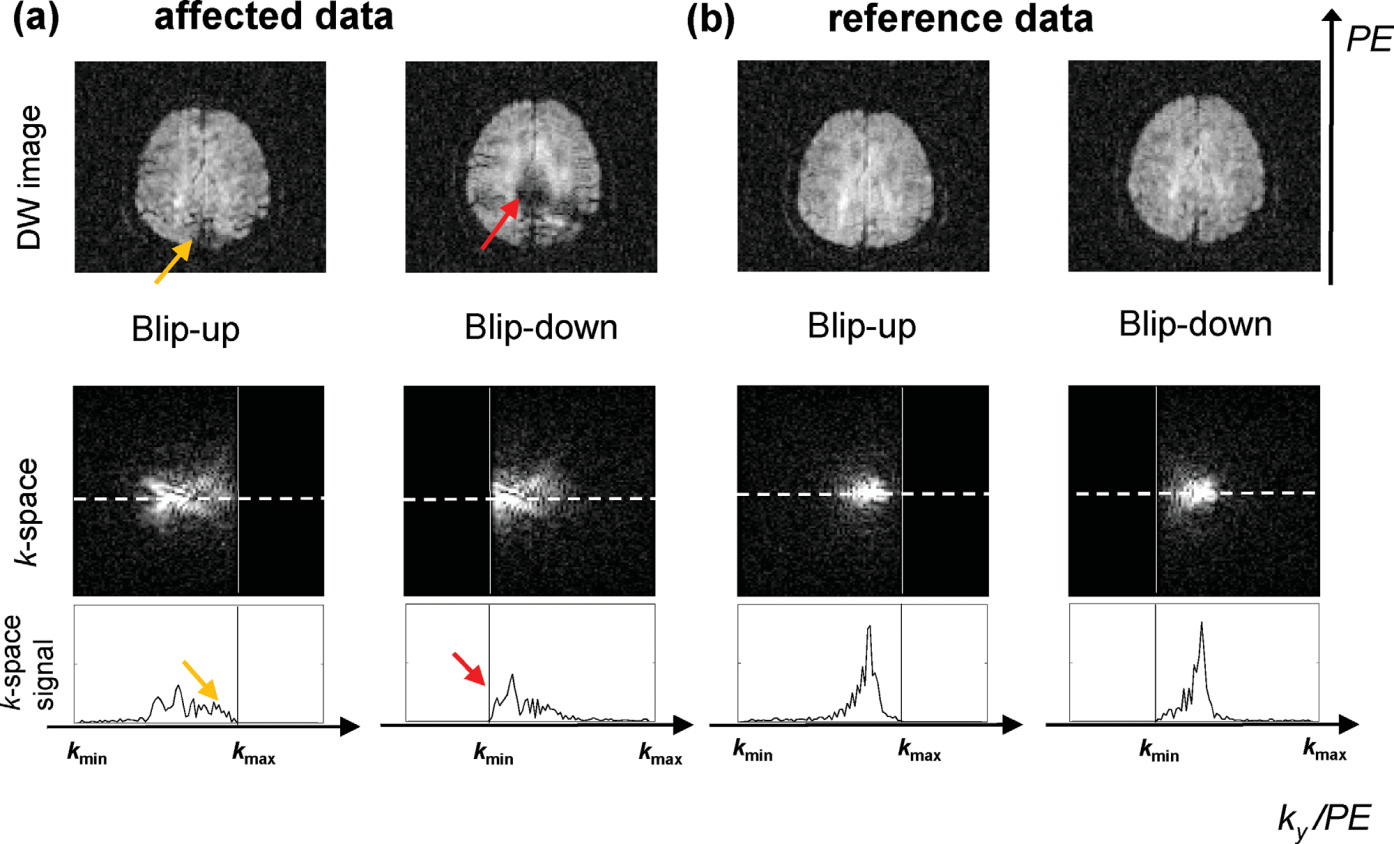
a : Example of signal-dropout due to vibration on an axial slice of a diffusion-weighted image (top row) as well as the corresponding shift in *k*-space (middle row, amplitude of complex raw data) and the projection of the *k*-space signal along the dashed line (bottom row). Severe signal-loss occurs (red arrow) if the echo center is shifted toward the shorter *k*-space edge (*k*_min_ for the blip-down data). As hypothesized, this signal-loss can be recovered if the PE direction is reversed (blip-up image). Note that the blip-up image shows also rudimentary signal-loss artifacts, however, in another region of the brain (yellow arrow) meaning that a partition of the echo is shifted towards *k*_max_, that is, the shorter *k*-space edge for the blip-up data. b: The reference data were acquired as in (a) but using a longer TR = 170 ms to reduce the vibration-induced echo shift effect. As expected, the diffusion-weighted images (top row) show no signal-dropout. Note that the geometrical distortions (due to susceptibility and eddy current effects) in blip-up and blip-down images (top row in a and b) have not been corrected to avoid interpolation artifacts. [Color figure can be viewed in the online issue, which is available at wileyonlinelibrary.com.]

Figure 3 shows the FA and the root-mean-square of the error of the tensor fit, and Fig 4 shows the quantified bias in FA. For dataset DTI^±^_1_ the vibration-induced bias in FA was visible in at least one dataset (blip-up or blip-down) of each subject (Fig 3a,b, arrows), while the extent of the bias varied between individuals (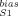 = 0.38; 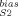 = 0.29; 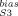 = 0.39; Fig 4). The artifact manifested itself in different regions for the blip-up (Fig 3a, yellow arrows) relative to the blip-down data (Fig 3b, red arrows). Averaged over subjects the standard arithmetic mean combination of blip-up and blip-down data reduced the vibration-induced bias in FA by 49% (from 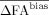
= 0.35 to 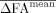
= 0.18, Fig 4). In contrast, the proposed COVIPER correction based on weighted-sum combination of blip-up and blip-down data reduced the error in FA by 72% 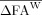
= 0.1) and the resulting maps showed better correspondence to the reference FA maps (Fig 3d,e). The contribution of miscellaneous effects that could not be attributed to vibration effects in the COVIPER method was about 6% (Fig 4). Note that the arithmetic-mean combination affects the tensor estimate for all regions showing vibration artifacts in the original blip-up or blip-down data, i.e., the union of affected regions in the blip-up and blip-down data. As a result the arithmetic-mean combination may show more widely spread artifacts (see regions highlighted by yellow and red arrows in Fig 3a–c), than any of the two original datasets (although usually at a lower level).

**Figure 3:**
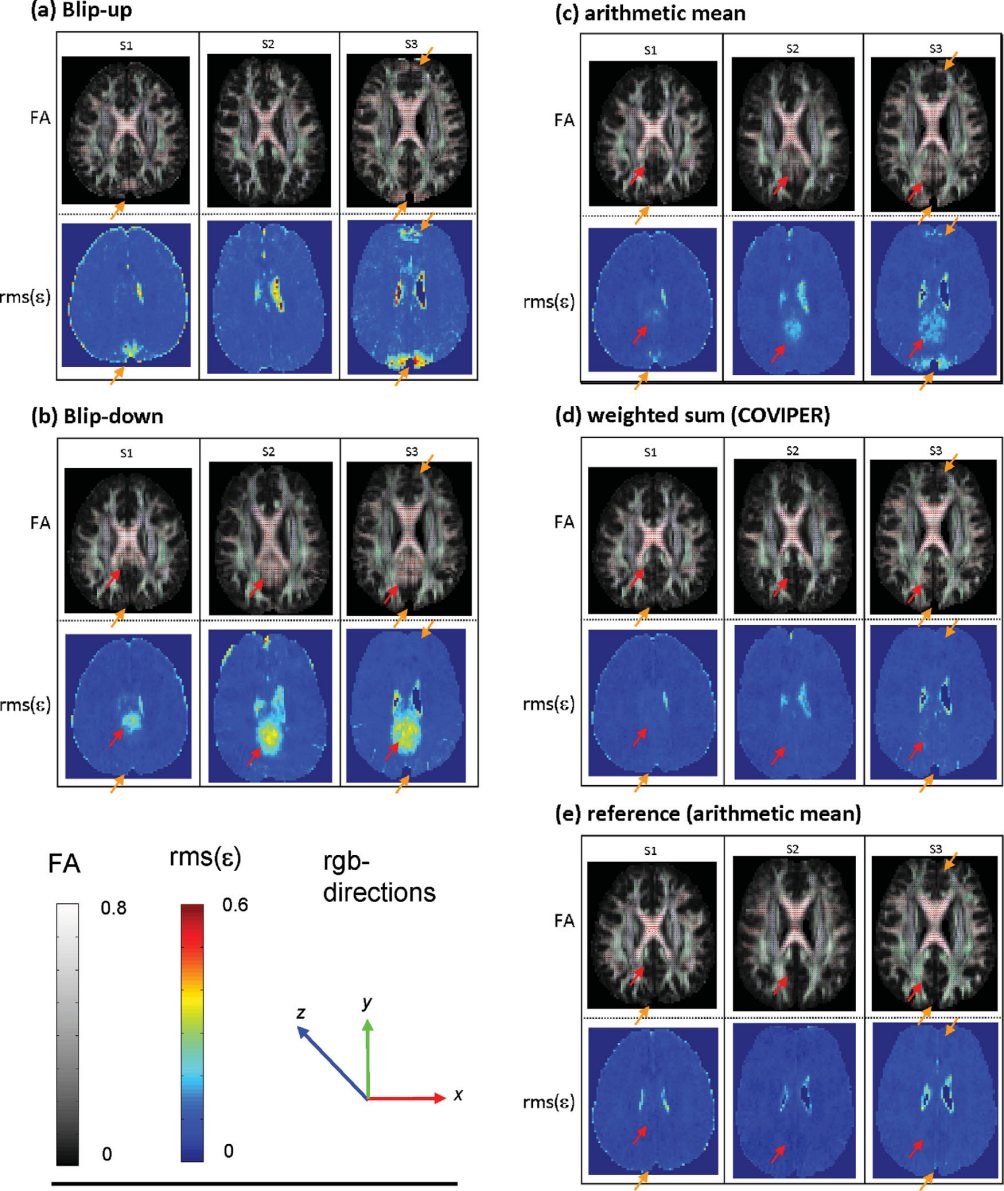
The FA (top row) in gray scale overlaid by the principled eigenvector in rgb-colors (red = *x*, green = *y,* and blue = z direction) and the root-mean-square of the error of the tensor fit rms(ε) in color (bottom row) using the DTI_1_^±^ data sets of subject S1–S3: (a) blip-up, (b) blip-down, (c) the arithmetic mean of blip-up and blip-down, (d) the weighted sum of blip-up and blip-down data (COVIPER), and (e) the arithmetic mean of blip-up and blip-down from the DTI_2_^±^ reference data. The vibration-induced bias in the FA maps (arrows) is clearly visible in the blip-up (a) and blip-down (b) data as well as in their arithmetic mean (c). The bias in FA is accompanied by an increased rms(ε) (arrows). COVIPER (d) reduced the bias in the FA maps and minimized the deviation from the reference FA map (e). Note that the location of the artifact is disjoint for the blip-up (yellow arrows) and blip-down (red arrows) data and it appears in similar regions across volunteers, although the extent and amplitude of the artifacts showed significant variation across volunteers. Furthermore, although less pronounced the artifact is visible in both locations (red and yellow arrows) when using the arithmetic mean combination (c).

**Figure 4:**
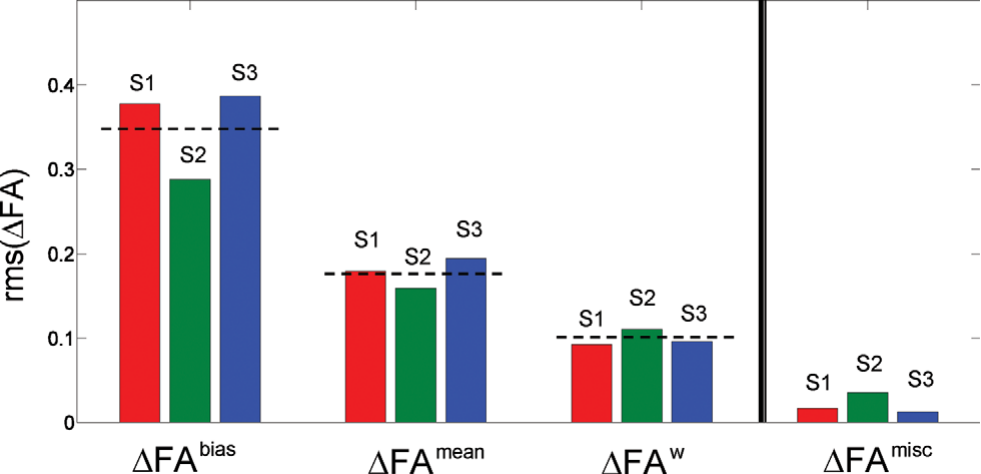
Quantification of the bias in FA for subjects S1–S3 using the root-mean-square FA-difference between affected (DTI_1_^±^) and reference (DTI_2_^±^) data within a region-of-interest based on: (a) the original data sets (ΔFA^bias^, Eq. 6a), (b) their arithmetic-mean combination (ΔFA^mean^, Eq. 6b), and (c) and their weighted-sum combination (ΔFA^W^, Eq. 6b). Furthermore, miscellaneous effects of the proposed correction method (ΔFA^misc^) were assessed using the root-mean-square FA difference between the arithmetic mean and weighted-sum combination of the reference data (Eq. 6c) containing negligible vibration artifacts. The region-of-interest was constructed based on the rms(ε) maps of the affected blip-up and blip-down data (DTI_1_^+^ and DTI_1_^−^). The subject-averaged FA differences (dashed lines) were = 0.35 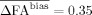
for the original data, = 0.18 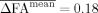
for the arithmetic-mean data, = 0.1 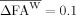
for the weighted-sum data. Accordingly, the FA bias of the original data was reduced by 49% using the arithmetic-mean combination (ΔFA^mean^), COVIPER (ΔFA^*w*^) leads to an improvement of 72%, and the contribution of miscellaneous effects (ΔFA^misc^) was 6%. [Color figure can be viewed in the online issue, which is available at wileyonlinelibrary.com.]

## DISCUSSION

We developed and validated a refined model and a correction of vibration artifacts in DTI using phase-encoding reversal (COVIPER). We acquired two DTI data sets with opposite PE direction (blip-up and blip-down data) and averaged them weighted by a function of the residual error of their tensor fit. Comparison with reference data containing negligible vibration artifacts showed that COVIPER efficiently reduced bias in FA due to vibration-induced signal loss. In contrast, the widely applied arithmetic mean of blip-up and blip-down data yields a smaller reduction in bias in FA and may even show more widely spread artifacts than any of the two original blip-up or blip-down datasets (though usually at a lower level).

The refined theoretical model for vibration-induced artifacts in DTI is based on the quantitative model of *k*-space shifts due to rigid body rotations (45, 46) and the qualitative description of non-rigid body brain-tissue movement due to vibration (23, 24). For axial echo-planar imaging acquisition with an anterior-posterior PE direction, the model predicts that non-rigid rotational in-plane movement of the brain-tissue and the strong diffusion gradients in *x* direction lead to the shift of the echo center (Eq. 2) in the PE direction. One consequence of this model is that signal-loss in Partial Fourier imaging might be recovered by inverting the PE direction (Fig 1). The successful correction of vibration artifacts using COVIPER supports this refined model (Figs 3 and 4).

Mechanical vibration, eddy currents and susceptibility differences are primary sources of artifacts in DTI (e.g., Ref.^20^). To enable cutting-edge neuroscience applications and facilitate clinical diagnosis, it is essential to minimize these image artifacts. It has been shown that eddy currents and susceptibility-induced artifacts can be retrospectively corrected using an arithmetic-mean combination of blip-up and blip-down DTI data (e.g., Refs.^23^, ^37^, ^42^, ^44^, and^57^). We showed that the arithmetic-mean combination should not be used in the presence of the vibration artifact. This is because the vibration artifact manifests itself in different regions for blip-up and blip-down data, when Partial Fourier imaging is used, as is common in DTI for reducing the echo time. The arithmetic-mean combination affects the tensor estimate for all regions showing vibration artifacts in the original data, i.e., the union of affected regions in blip-up and blip-down images, resulting in FA bias in previously unaffected regions (Fig 3). Here, we proposed to reduce the vibration artifact using a weighted-average that reduces the contribution of the data affected by the vibration artifact as estimated from the error in the tensor fit. The weighted-average combination showed the smallest bias in FA and the best correspondence to reference DTI data, with an average reduction of 72% in the residual error in FA (Fig 4).

In this study, we used zero filling for Partial Fourier image reconstruction. However, the signal-dropout artifact due to vibration might be further distributed across space if other Partial Fourier image reconstruction methods such as homodyne reconstruction were used (45).

The performance of the suggested correction method will depend on the extent of the shift of the echo center; if the shift is too large, i.e., the shift is in the same order of magnitude as the longest extent of the *k*-space window (Fig 1), no signal will be recovered. To overcome this potential problem (which we did not observe), the analogy between echo shifts due to susceptibility artifacts (38, 48) and vibration artifacts could be further exploited by applying other established techniques for reducing signal loss in gradient echo echo-planar imaging such as *y*-shim gradient prepulses (38) or appropriate slice tilts (49, 58).

To save scan time, cardiac triggering was not used in this study. However, we expect the cardiac pulsation artifacts (see e.g., Refs.^21^ and^30^) to be minor compared to the vibration artifacts in the short TR data (DTI1), because most of the residual error of the tensor fit could be explained by the vibration artifact (Fig 3). However, the COVIPER method can also be combined with cardiac triggered data. However, it must be noted that the first slice after each trigger pulse is acquired with an increased slice TR (i.e., due to waiting for the trigger and avoiding systole). Because one of the remedies recommended to avoid vibrations induced artifacts is expanded TR (23) cardiac triggering is expected to be partially beneficial in this respect.

One might argue that the residual error of the estimated diffusion tensor fit, which itself is affected by the vibration artifact, is not the optimal choice for the weighting process (i.e., to assess data quality) because there is a certain circularity in the reasoning. Despite the problem of circularity, previous studies successfully used the residual error of the tensor fit to assess and correct perturbed DTI data. It was used, e.g., to detect the improvement of data quality after correcting for eddy currents (35, 59), or to down-weight regions with poor data quality to estimate local perturbation fields (60), or to detect regions affected by the vibration artifact (23).

We used a field map based correction (50) for susceptibility-induced distortions, to geometrically match blip-up and blip-down data. Although it has been shown that the field map based correction can be applied to DTI data (25, 37), the correction may be finessed by methods directly exploiting the information in the PE reversed images (23, 37, 42, 44, 57).

## CONCLUSION

We have presented a new correction method for vibration artifacts that uses two DTI data sets with reversed phase-encoding direction (blip-up and blip-down) to recover vibration-induced signal-loss in Partial Fourier imaging. This method can be readily combined with previously suggested correction methods for vibration artifacts (23, 25, 27) as well as with those for eddy currents and susceptibility-induced distortion (e.g., Refs.^37^, ^41^, and^44^), which are also based on DTI data acquired with PE reversal. The combination of these different approaches is an important future direction of research towards providing DTI with minimal artifacts.
